# Exploring the relationship between spousal violence during pregnancy and subsequent postpartum spacing contraception among first-time mothers in India

**DOI:** 10.1016/j.eclinm.2020.100414

**Published:** 2020-06-15

**Authors:** Lotus McDougal, Jay G. Silverman, Abhishek Singh, Anita Raj

**Affiliations:** a Center on Gender Equity and Health, School of Medicine, University of California San Diego, 9500 Gilman Drive, La Jolla, CA 92093, USA; b Department of Public Health & Mortality Studies, International Institute for Population Sciences, Govandi Station Road, Deonar, Mumbai 400 088, India

**Keywords:** Postpartum contraception, Abuse during pregnancy, Gender-based violence, Male-controlled contraception, Female-controlled contraception, Spacing contraception

## Abstract

**Background:**

There is a growing body of research exploring how intimate partner violence affects contraceptive decision-making, recognizing that these decisions are reflective not only of access and acceptability, but also spousal power imbalances. Unfortunately, there is a dearth of knowledge regarding contraceptive choices following gender-based violence during pregnancy. There are an estimated 7·8 million in India affected by violence during pregnancy, and an ongoing, heavy reliance on female sterilization as the dominant form of contraception. This study examines the relationship between abuse during pregnancy and subsequent postpartum spacing contraception in India.

**Methods:**

This analysis used cross-sectional, nationally representative data from first-time mothers of children aged 6–48 months in India. Multinomial regression models assessed relationships between spousal physical violence during pregnancy and postpartum spacing contraception (none, female-controlled, male-controlled).

**Findings:**

Two percent of first-time mothers (2·4%) reported spousal physical violence while pregnant. Women who reported abuse during pregnancy were less likely to subsequently use male-controlled contraception than no contraception (adjusted relative risk ratio [aRRR]=0·3, 95% CI 0·1–0·8; *p* = 0·02) and more likely to use female-controlled vs. male-controlled spacing contraception (aRRR=7·5, 95% CI 2·1–25·4, *p*<0·01).

**Interpretation:**

Women who experience spousal abuse during pregnancy have different postpartum contraceptive use patterns. The unique needs of this population should be incorporated into antenatal and postpartum contraceptive counseling. Efforts to increase spacing contraception use in India must consider experiences of gender-based violence and coercion.

**Funding:**

This work was supported by the 10.13039/100000865Bill & Melinda Gates Foundation [OPP1179208, PI: Raj]. Funders had no role in the design, analysis or interpretation of this research.

## 1 Introduction

Gender-based violence is a widespread and serious abuse of human rights that affects more than one in three women globally [1]. This violence is sustained by inequitable gender norms and values, as well as power imbalances, and can lead to serious adverse health consequences [1], [2], [3], [4], [5]. Research from multiple settings suggests that women who experience gender-based violence from an intimate partner tend to have lower utilization of reproductive health services, often including decreased use of contraceptives that require the cooperation of male partners [[5], [6], [7], [8]].

Women who experience gender-based violence while pregnant, which is often perpetrated by a spouse, face a double barrel of vulnerability, with risk of harm to both themselves and their pregnancies [3, [9], [10], [11]]. Recent estimates suggest that in India, a country bearing heavy burdens of gender inequities and violence, more than 9 million women (3·9% of reproductive-aged mothers) have experienced violence during their pregnancies [[11], [12], [13], [14], [15], [16]].

There is a serious, but heterogeneous, relationship between intimate partner violence and contraceptive use in India and elsewhere [[17], [18], [19], [20]]. Evidence of differential contraceptive choices in circumstances of spousal abuse suggests that women who have experienced violence may have a higher reliance on non-partner dependent (e.g. female-controlled) contraceptive methods, many of which can be used covertly (e.g., pill, IUD) [17]. Understanding contraceptive decision-making and utilization not only in terms of effectiveness, but also through the lens of partner control over utilization, is a key component of appropriate support, screening and health service delivery to victims of violence [8,19]. This distinction is particularly important in India, where more than half of married women have spouses who exhibit some form of controlling behavior, one in three married women have experienced some form of intimate partner violence, and the most common form of spacing contraception is condoms (9%), a method dependent on male partner cooperation [11].

Understanding the relationship between spousal violence during pregnancy and subsequent postpartum contraceptive use in India is important not only to support healthy pregnancy spacing, but also to ensure that the method mix is able to support the potentially differential needs of women who have, and have not, experienced spousal violence during pregnancy. This information can guide health care protocols to ensure that contraceptive counseling offers information relevant to women who may have experienced spousal violence during pregnancy, particularly in the context of postnatal care. This paper aims to deepen understanding of this question in India, with a goal of identifying areas where access to appropriate contraceptive methods can be improved to ensure equitable options for all women.

## 2 Methods

We used data from India's 2015–16 National Family Health Survey (NFHS-4), a nationally representative survey of demographic, health and social indicators. NFHS-4 used a two-stage cluster sampling approach, details of which have been published elsewhere [11]. All women aged 15–49 in selected households were eligible to participate; verbal consent was obtained from women who agreed to be interviewed. In total, 699,686 women were interviewed in person by female enumerators, with a response rate of 97%. A subset of these women (one woman per household in 15% of households) responded to questions on violence (*n* = 79,729). This sample was designed to provide representative estimates of violence against women at the state and national levels.

The primary predictor of interest was spousal physical violence during pregnancy, assessed by the questions “Has any one ever hit, slapped, kicked, or done anything else to hurt you physically while you were pregnant?” and if yes, “Who has done any of these things to physically hurt you while you were pregnant? Anyone else?”. Women were considered to have experienced spousal physical violence during pregnancy if they reported abuse during pregnancy that was perpetrated by a current or former husband, and to not have experienced spousal physical violence during pregnancy if they responded “no” to the first question. To account for the “ever/never” phrasing of the abuse during pregnancy question, this analysis included women with only one living child who did not report any previous pregnancies that did not result in a live birth, to ensure that reported experiences of abuse during pregnancy were specific to the index pregnancy. The analytic sample was further restricted to women who gave birth 6–48 months prior to interview (to allow time for initiation of postpartum contraception), were currently married and had no missing responses (*n* = 2856).

Our outcome variable was postpartum spacing contraception, assessed as the first type of spacing contraception initiated subsequent to the index pregnancy. This was categorized as none, female-controlled methods (pill, IUD, injections, lactational amenorrhea), or male-controlled methods (condom, periodic abstinence/rhythm, withdrawal).

In order to isolate the association between spousal physical violence during pregnancy and postpartum spacing contraception, regression models adjusted for key covariates related to the index birth, as well as social and gender equity factors associated with these measures in past research [17,21,22]. Measures related to the index birth included mother's receipt of family planning advice from a health worker (none, health worker meeting without family planning advice, health worker meeting with family planning advice), father's receipt of family planning advice from a health worker during wife's pregnancy (no, yes), delivery location (home, public facility, private facility/NGO/trust hospital), sex of index child (female, male), maternal postpartum check (none, yes and within two days of birth, yes but more than two days since birth), and months since the index child's birth. Deliveries were considered to have occurred at home if they took place in any home (respondent's, parent's or other). Public delivery facilities included government or municipal hospitals, government dispensaries, urban health centers, urban health posts, urban family welfare centers, community health centers, rural hospitals, primary health centers, sub-centers (the first point of contact with the public, primary health system in India) and other public facilities. Private/NGO/trust hospital delivery facilities included private hospitals or maternity homes or clinics, other private sector health facilities and NGO or trust hospitals and clinics. Social and gender equity measures included respondent's age (in years), household wealth, education (in years), and age at first marriage or cohabitation (<18 years, ≥18 years). Household wealth is a continuous variable ranging between 0–1 (with 0 being the poorest households and 1 being the wealthiest households) that represents the relative wealth of a household as determined by a principal components analysis of household wealth and assets [23].

As the outcome variable had three discrete, unordered levels, multinomial regression models were used. Multinomial regression models compare different outcome levels against a selected base, or reference, category, and can produce relative risk ratios, which are ratios of the relative risk of a given outcome as compared to the relative risk of the reference outcome. This analysis assessed associations between physical violence during the index pregnancy (yes/no), and postpartum spacing contraception. Models compared male-controlled vs. none, female-controlled vs. none, and female-controlled vs. male-controlled spacing contraception to better understand dynamics across groups, adjusting for measures related to the index pregnancy/birth as well as social and gender equity. All analyses were conducted using Stata SE 16·1.

Ethical approval for data collection was provided by IRBs for the International Institute for Population Sciences and ICF. Ethical exemption for analysis of this deidentified, publicly available data was provided by the University of California San Diego IRB.

### 2.1 Role of funding

Funders had no role in study design, collection, analysis, or interpretation of data, the writing of this manuscript, or the decision to submit the paper for publication.

## 3 Results

Two percent of first-time mothers in this sample (2·4%) experienced spousal physical violence while they were pregnant (Table 1). Half of women (49%) did not use any postpartum spacing contraception in the window between giving birth and being interviewed (an average of 21 months). One in three mothers (34%) first used a male-controlled spacing contraception (51% condom, 27% periodic abstinence/rhythm, 22% withdrawal), and 18% first used female-controlled spacing contraception (63% pill, 27% IUD, 3% injections, 7% lactational amenorrhea) as the first postpartum method (Table 2). In contrast to the overall analytic sample, among women who experienced spousal physical abuse during pregnancy, the prevalence of initiating female-controlled postpartum contraception was higher than the prevalence of initiating male-controlled postpartum contraception (4·9% vs. 0·7%; Table 1). Contraceptive use began an average of six months postpartum, and at the time of interview, 83% of mothers were still using the first form of contraception initiated postpartum (Table 2).

**Table 1:** Descriptive summary of postpartum spacing contraceptive use, spousal abuse during pregnancy and covariates among primiparous mothers of children aged 0–3 years in India, 2015–16.

			Postpartum spacing contraceptive use					
	Total		None		Male-controlled^1^		Female-controlled^2^	
	n	% (95% CI)	n	%	n	%	n	%
Total^3^	2856	100	1408	48·9 (45·7–52·1)	947	33·5 (30·1–37·1)	501	17·6 (15·4–19·9)
Spousal abuse during pregnancy								
None	2808	97·6 (96·5–98·4)	1384	97·4 (95·9–98·4)	938	99·3 (98·4–99·7)	486	95·1 (89·6–97·8)
Any	48	2·4 (1·6–3·5)	24	2·6 (1·6–4·2)	9	0·7 (0·3–1·6)	15	4·9 (2·2–10·4)
**Index birth**								
Mother received family planning advice from health worker in last 3 months of pregnancy								
None	1316	46·7 (43·5–49·8)	697	49·9 (45·6–54·1)	420	48·5 (42·0–55·1)	199	34·1 (27·8–40·9)
Health worker meeting without family planning advice	429	14·5 (12·7–16·5)	220	15·7 (13·0–18·8)	148	13·2 (10·4–16·6)	61	14·0 (9·4–20·3)
Health worker meeting with family planning advice	1111	38·8 (36·1–41·7)	491	34·5 (30·8–38·3)	379	38·3 (32·6–44·4)	241	52·0 (45·3–58·5)
Father received family planning advice from health worker during wife's pregnancy								
No	1028	34·2 (31·0–37·5)	552	36·8 (33·0–40·8)	330	35·9 (29·3–43·0)	146	23·6 (18·0–30·3)
Yes	1828	65·8 (62·5–69·0)	856	63·2 (59·2–67·0)	617	64·1 (57·0–70·7)	355	76·4 (69·7–82·0)
Delivery location								
Home	268	7·6 (6·2–9·2)	160	7·1 (5·6–9·1)	69	6·6 (4·1–10·3)	39	10·7 (6·3–17·5)
Public facility	1708	52·4 (49·4–55·3)	806	50·8 (46·6–55·1)	542	49·2 (43·6–54·8)	360	62·8 (55·7–69·4)
Private facility/NGO/ trust hospital	880	40·1 (37·3–42·9)	442	42·0 (37·9–46·3)	336	44·3 (38·7–49·9)	102	26·5 (20·5–33·7)
Sex of index child								
Female	1358	49·3 (46·2–52·5)	690	49·3 (45·2–53·5)	436	49·7 (42·7–56·7)	232	48·8 (41·1–56·5)
Male	1498	50·7 (47·5–53·8)	718	50·7 (46·5–54·8)	511	50·3 (43·3–57·4)	269	51·3r (43·6–58·9)
Maternal postpartum check								
None	612	20·4 (17·9–23·1)	373	23·8 (20·1–28·0)	149	15·8 (12·4–19·9)	90	19·5 (14·4–26·0)
≤2 days since birth	2060	72·6 (69·8–75·3)	939	69·6 (65·4–73·5)	738	77·7r (72·7–82·0)	383	71·4 (63·9–78·0)
>2 days since birth	184	7·0 (5·5–8·7)	96	6·6 (5·0–8·6)	60	6·5 (4·0–10·3)	28	9·0 (5·2–15·2)
Months since birth of index child^4^		21·4 (11·5)		19·7 (11·4)		22·2 (11·2)		24·7 (11·5)
**Social and gender equity**								
Age (years) 4		23·9 (3·9)		24·0 (4·2)		24·0 (3·7)		23·5 (3·4)
Household wealth^4^^,^^5^		0·4 (1·0)		0·3 (0·9)		0·6 (1·0)		0·2 (1·0)
Education (years) 4		9·6 (4·7)		9·2 (4·9)		10·2 (4·6)		9·3 (4·2)
Age at first marriage/cohabitation								
<18 years	561	23·2 (20·8–25·7)	306	23·9 (20·7–27·4)	150	19·9 (15·8–24·7)	105	27·5 (21·3–34·7)
≥18 years	2295	76·8 (74·3–79·2)	1102	76·1 (72·6–79·3)	797	80·1 (75·4–84·2)	396	72·5 (65·3–78·7)

Note: Sample sizes are unweighted, percentages are weighted.

1 Condom, periodic abstinence/rhythm, withdrawal.

2 Pill, IUD, injections, lactational amenorrhea.

3 Row percents. All other percents are column.

4 Mean (SD).

5 Values of 0 represent the poorest households, and values of 1 represent the wealthiest households.

**Table 2:** Prevalence and characteristics of male vs. female controlled postpartum contraception for first postpartum contraceptive use among primiparous mothers of children aged 0–3 years in India, 2015–16.

	Total		Male-controlled		Female-controlled	
	n	% (95% CI)	n	% (95% CI)	n	% (95% CI)
First postpartum contraceptive method						
Pill					314	63·1 (56·6–69·2)
IUD					142	26·7 (21·5–32·5)
Injection					15	3·0 (1·2–7·3)
Lactational amenorrhea					30	7·3 (4·3–12·0)
Condom			499	50·9 (44·5–57·3)		
Periodic abstinence/rhythm			197	27·1 (20·6–34·8)		
Withdrawal			251	22·0 (17·9–26·7)		
Months since birth of initiation of first postpartum contraception^1^		5·8 (4·9)		5·6 (4·5)		6·1 (5·6)
Percent of first postpartum contraceptive usage that was ongoing at time of interview (no discontinuation)	1174	83·0 (79·8–85·8)	783	85·2 (81·4–88·4)	391	78·6 (72·6–83·7)

Note: Sample sizes are unweighted, percentages and means are weighted.

1 Mean (SD).

Multivariable models show that women who experienced abuse during their index pregnancy were less likely to use initiate male-controlled contraception than no postpartum spacing contraception (aRRR=0·31, 95% CI 0·11–0·83; *p* = 0·02), and more likely to initiate a female-controlled method (aRRR=7·49, 95% CI 2·12–25·35; *p*<0·01) than to use a male-controlled method (Table 3). There was no difference in the likelihood of initiating female-controlled vs. no spacing contraception based on experiences of abuse during pregnancy. Antenatal family planning counseling for either spouse increased the likelihood of initiating female-controlled contraception postpartum relative to male-controlled contraception (wife aRRR=1·57, 95% CI 1·04–2·37; *p* = 0·03; husband aRRR=2·06, 95% CI 1·34–3·16; *p*<0·01) and to no contraception (wife aRRR= 1·82, 95% CI 1·26–2·64; *p*<0·01; husband aRRR=1·86, 95% CI 1·25–2·76; *p*<0·01). Women who delivered in private facilities were less likely than those who delivered in public facilities to initiate female-controlled postpartum contraception, relative to both no postpartum spacing contraception and male-controlled postpartum spacing contraception (female-controlled vs. none aRRR=0·52, 95% CI 0·35–0·77; *p*<0·01 and female-controlled vs. male-controlled aRRR=0·67, 95% CI 0·45–0·98; *p* = 0·04).

**Table 3:** Multinomial multivariable regressions showing associations between spousal violence during pregnancy and postpartum spacing contraception use among primiparous mothers of children aged 0–3 years in India, 2015–16.

	Male-controlled spacing contraception^1^ (reference: no postpartum spacing contraception		Female-controlled^2^ spacing contraception (reference: no ``postpartum spacing contraception)		Female-controlled^2^ spacing contraception (reference: male-controlled spacing contraception^1^)	
	aRRR (95% CI)	p-value	aRRR (95% CI)	p-value	aRRR (95% CI)	p-value
Spousal abuse during pregnancy						
None	REF		REF		REF	
Any	0·31 (0·11–0·83)	0·02	2·30 (0·85 −6·23)	0·10	7·49 (2·12 – 25·35)	<0·01
**Index birth**						
Mother received family planning advice from health worker in last 3 months of pregnancy						
None	REF		REF		REF	
Health worker meeting without family planning advice	0·97 (0·66–1·44)	0·89	1·13 (0·68–1·88)	0·64	1·16 (0·66–2·05)	0·61
Health worker meeting with family planning advice	1·16 (0·83–1·62)	0·37	1·82 (1·26–2·64)	<0·01	1·57 (1·04–2·37)	0·03
Father received advice from health provider/worker on family planning during wife's index pregnancy						
No	REF		REF		REF	
Yes	0·91 (0·64–1·27)	0·57	1·86 (1·25–2·76)	<0·01	2·06 (1·34 – 3·16)	<0·01
Delivery location						
Home	1·40 (0·79–2·47)	0·25	1·40 (0·69–2·84)	0·35	1·00 (0·44–2·26)	0·99
Public facility	REF		REF		REF	
Private facility/NGO/trust hospital	0·78 (0·57–1·07)	0·12	0·52 (0·35–0·77)	<0·01	0·67 (0·45–0·98)	0·04
Sex of index child						
Female	REF		REF		REF	
Male	1·05 (0·76–1·45)	0·78	1·08 (0·76–1·54)	0·66	1·03 (0·69–1·55)	0·88
Maternal postpartum check						
None	REF		REF		REF	
≤2 days since birth	1·59 (1·05–2·41)	0·03	1·34 (0·84–2·15)	0·22	0·84 (0·53–1·35)	0·48
>2 days since birth	1·32 (0·72–2·40)	0·37	1·40 (0·62–3·15)	0·42	1·06 (0·45–2·50)	0·89
Months since birth of index child	1·02 (1·01–1·04)	<0·01	1·05 (1·03–1·07)	<0·01	1·03 (1·01–1·05)	<0·01
**Social and gender equity**						
Age (years)	0·94 (0·91–0·98)	<0·01	0·92 (0·87–0·97)	<0·01	0·97 (0·92–1·03)	0·34
Household wealth	1·46 (1·21–1·76)	<0·01	0·89 (0·72–1·10)	0·27	0·61 (0·48–0·78)	<0·01
Education (years)	1·01 (0·97–1·05)	0·58	1·04 (0·99–1·09)	0·13	1·03 (0·98–1·08)	0·32
Age at first marriage/cohabitation						
<18 years	0·80 (0·55–1·15)	0·22	0·81 (0·53–1·25)	0·35	1·02 (0·64–1·63)	0·93
≥18 years	REF		REF		REF	

Note: Results control for all variables shown.

1 Condom, periodic abstinence/rhythm, withdrawal.

2 Pill, IUD, injections, lactational amenorrhea.

## 4 Discussion

While half of first-time mothers in India did not use postpartum contraception, among those who did, initiating male-controlled methods was more common than initiating female-controlled methods. More than two percent of women in this sample reported spousal physical abuse during pregnancy. At current population estimates, there are thus an estimated 2·8 million first-time Indian mothers of young children who have been physically abused by their husbands while pregnant [12]. Women who reported physical violence during their pregnancy were more likely to both not use postpartum contraception and to initiate female-controlled spacing contraception than to initiate male-controlled postpartum contraception, as compared to women not abused during pregnancy.

Previous research in India has shown a divergent associations between marital sexual violence and different forms of spacing contraception, and inconsistent associations for marital physical violence [17,20]. As abuse during pregnancy was assessed as “physical hurt”, this suggests that abuse during this vulnerable period may have unique contraceptive ramifications distinct from more generalized spousal physical abuse, and that more conventional categorizations of contraceptive use such as modern/traditional or spacing/limiting may not adequately capture the aspects of contraceptive use most important to victims of violence. As has been indicated by others, examining contraceptive use in terms of control appears necessary to more completely understand the choices and utilization patterns of women who have experienced abuse [8,19,24].

The contraceptive groupings used in this study (male- and female-controlled) are important, but not comprehensive, lenses with which to look at the relationship between violence during pregnancy and subsequent contraceptive use. Other factors, including effectiveness, covert use, accessibility and acceptability, also likely influence the relationship between spousal abuse during pregnancy and postpartum contraception. We were unable to explore these other potential confounders due to both a lack of data in these areas in our dataset, as well as small sample sizes among postpartum contraceptive users who reported spousal violence during pregnancy. These other factors that may influence postpartum contraceptive use differentially for women who were and were not abused during their pregnancies merit further study, and our results should be interpreted within the context of these caveats.

These results underscore the need to provide contraceptive counseling, both antenatal and postpartum, that acknowledges that women facing abuse may want a female-controlled, less detectable method. Screening for violence, while necessary, may not find the majority of women who experience spousal violence. Nearly eighty percent of women in India who experienced violence from their current husbands never told *anyone* about this abuse; that low level of disclosure is similar elsewhere. [11,25] This counseling, which should be predicated by the availability of a diverse contraceptive method mix, should be sensitive to highlighting a number of features of the available contraceptive types, including effectiveness and potential side effects, but also ease of use and control. Such counseling may increase contraceptive use and provide a critical opportunity for providers to support and assist women facing abuse from their partner or family. Indeed, women's and men's receipt of antenatal family planning counselling was strongly associated with postpartum initiation of female-controlled contraception, a relationship that was unchanged after adjusting for abuse during pregnancy (results not shown), suggesting there is room for further tailoring of this advice. There is a clear disparity in postpartum contraceptive uptake in public vs. private facility deliveries, indicating that private facilities may benefit from increasing postpartum contraceptive counseling availability and frequency prior to discharge [26].

This study uses cross-sectional data, and thus cannot assume causality, though the temporality of our variables suggests it is possible. Self-report data are subject to recall and social desirability bias. Findings may have limited generalizability to first-time mothers in India, but this is a large and important population. Spousal abuse during pregnancy has a relatively low prevalence (2·4% in this sample), which should be considered when interpreting results. Finally, to focus on spacing contraception, we excluded the 1% of first-time mothers in the sample who reported postpartum sterilization.

Postpartum contraception, a critical component of empowering women to achieve their desired fertility, is informed by the reproductive and personal history of women. Identifying victims of abuse and offering safe and effective contraceptive options is an important step in improving contraceptive access and availability for all women.

## Declaration of Competing Interests

All authors received grant funding from the Bill and Melinda Gates Foundation supporting the analysis and writing of this manuscript (OPP1179208, PI: Raj). Funders had no role in the design, analysis or interpretation of this research.
